# Endogenous Generation and Signaling Actions of Omega-3 Fatty Acid Electrophilic Derivatives

**DOI:** 10.1155/2015/501792

**Published:** 2015-08-03

**Authors:** Chiara Cipollina

**Affiliations:** ^1^Fondazione Ri.MED, Palermo, Italy; ^2^Istituto di Biomedicina e Immunologia Molecolare (IBIM), Consiglio Nazionale delle Ricerche, 90146 Palermo, Italy

## Abstract

Dietary omega-3 polyunsaturated fatty acids (PUFAs) are beneficial for a number of conditions ranging from cardiovascular disease to chronic airways disorders, neurodegeneration, and cancer. Growing evidence has shown that bioactive oxygenated derivatives are responsible for transducing these salutary effects. Electrophilic oxo-derivatives of omega-3 PUFAs represent a class of oxidized derivatives that can be generated via enzymatic and nonenzymatic pathways. Inflammation and oxidative stress favor the formation of these signaling species to promote the resolution of inflammation within a fine autoregulatory loop. Endogenous generation of electrophilic oxo-derivatives of omega-3 PUFAs has been observed in *in vitro* and *ex vivo* human models and dietary supplementation of omega-3 PUFAs has been reported to increase their formation. Due to the presence of an *α*,*β*-unsaturated ketone moiety, these compounds covalently and reversibly react with nucleophilic residues on target proteins triggering the activation of cytoprotective pathways, including the Nrf2 antioxidant response, the heat shock response, and the peroxisome proliferator activated receptor *γ* (PPAR*γ*) and suppressing the NF-*κ*B proinflammatory pathway. The endogenous nature of electrophilic oxo-derivatives of omega-3 PUFAs combined with their ability to simultaneously activate multiple cytoprotective pathways has made these compounds attractive for the development of new therapies for the treatment of chronic disorders and acute events characterized by inflammation and oxidative stress.

## 1. Introduction

Dietary intake of omega-3 polyunsaturated fatty acids (PUFAs) has been associated with beneficial effects for human health, leading to a reduced cardiovascular risk both in primary and in secondary prevention, contrasting systemic inflammation and neurodegeneration as well as the development of chronic disorders including cancer and inflammatory airways diseases [1–4]. Upon dietary intake, omega-3 PUFAs are readily incorporated into lipid membranes and modify cellular signaling through multiple mechanisms. Omega-3 fatty acid membrane enrichment occurs mainly at expense of arachidonic acid (AA), resulting in reduced production of AA-derived proinflammatory prostaglandins and leukotrienes [5]. A second mechanism of action is related to the high degree of unsaturation of omega-3 PUFAs which results in altered membrane fluidity and leads to the disruption of lipid raft-related proinflammatory signaling [6, 7]. In addition to these established mechanisms, in the last decade a third mechanism of action has emerged related to the conversion of omega-3 PUFAs into oxygenated bioactive derivatives to promote the resolution of inflammation. Once incorporated into cell membranes, omega-3 PUFAs become available for conversion into bioactive oxidized derivatives. Consistently, dietary intake of omega-3 PUFAs significantly enhances the production of omega-3 PUFA derived oxidized species [8–11]. Formation of bioactive oxygenated derivatives of omega-3 PUFAs occurs via enzymatic and nonenzymatic pathways and uses both free and esterified fatty acids as substrates. In particular, electrophilic oxo-derivatives are generated during oxidative reactions and represent a recently discovered class of bioactive omega-3 PUFAs. These species are released by the cell during oxidative stress and inflammation to exert autocrine and paracrine signaling. Omega-3 PUFAs electrophilic derivatives appear to be the active mediators that transduce the beneficial actions observed upon dietary administration of omega-3 PUFAs and therefore there has been a growing interest in characterizing their formation and signaling actions in health and disease. While originally viewed as toxic mediators, these compounds have recently been appreciated for their anti-inflammatory role and oxidative stress suppression through the expression of phase II genes. These actions are triggered by the covalent reaction of electrophilic PUFAs with nucleophilic residues on target proteins leading to the activation of several cytoprotective pathways. Due to their endogenous nature and ability to simultaneously activate multiple signaling pathways, these electrophilic compounds have attracted great attention for the development of new drugs for the treatment of diseases characterized by inflammation and oxidative stress [12–18].

## 2. Chemistry of Electrophilic Lipids

**Figure 1:**
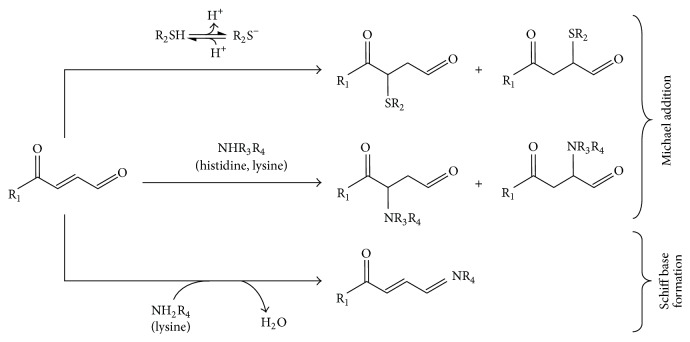
Reaction scheme of electrophilic lipid derivatives. Electrophilic *α*,*β*-unsaturated ketone moieties react with nucleophilic residues on target proteins (thiolates of cysteines and amino groups of histidine and lysine) via Michael reaction. In the case of bifunctional electrophiles, the aldehyde group reacts with primary amines of lysine generating Schiff base adducts.

Enzymatic and nonenzymatic oxidation of omega-3 PUFAs generates a broad range of oxygenated species containing electrophilic *α*,*β*-unsaturated ketone moieties. The presence of a double bond conjugated to a ketogroup renders the *β*-carbon electron poor and is therefore susceptible to nucleophilic attack. Reaction of *α*,*β*-unsaturated ketones with nucleophiles occurs via Michael addition during which the electron-poor *β*-carbon accepts the pair of electrons of the electron-rich nucleophile forming a covalent bond (Figure 1). The chemistry governing the reaction between electrophiles and nucleophiles is described by the hard/soft acid-base theory [19] that provides a framework for understanding the reactivity of these species in which soft (polarizable) electrophiles preferentially react with soft nucleophiles while hard (nonpolarizable) electrophiles favorably react with hard nucleophiles. The “electrophilicity index” was later introduced by Parr et al. to better describe the chemical properties of electrophilic species [20]. The electrophilicity index combines softness and chemical potential and can be used to predict the reactivity of an electrophile and to anticipate its biological activity and potential toxicity [21, 22]. For example, several mutagenic compounds present a high electrophilicity index and are hard electrophiles thus reacting more favorably with hard nucleophilic groups found in purine and pyrimidine bases leading to irreversible modification of DNA [23]. In contrast, *α*,*β*-unsaturated ketones are soft electrophiles that preferentially react with soft nucleophiles, including cysteine thiols and to a lesser extent primary and secondary amines of lysine and histidine residues, respectively. More specifically, the thiolate anion form of cysteine is the preferred target for *α*,*β*-unsaturated ketones [24, 25]. In this regard, the pKa of a cysteine is defined as the pH at which 50% is in an ionized state (deprotonated) and is between 8 and 9 for most biologically relevant thiols, close to the physiological pH range. This means that small changes of cysteine pKa that can be caused by conformational modifications, changes of intracellular distribution, or protein-lipid interaction will significantly affect thiolate levels. This modulation of cysteine reactivity provides a framework for fine-tuning of posttranslational modifications within physiological pH ranges [25]. In addition to cysteine pKa, the reactivity of a given electrophile towards a nucleophilic residue will depend on structural factors including the accessibility of the nucleophilic site and the presence of a microenvironment that stabilizes protein-lipid interaction thus favoring Michael addition. Polar and hydrophobic interactions between the electrophilic fatty acid and exposed amino acids are crucial for the right positioning of the reactive carbon in order for the Michael addition to occur. In this respect, extensive structural investigations on the covalent binding between electrophilic lipids (oxo-fatty acids and nitroalkenes) and Cys-285 within the ligand binding pocket of the peroxisome proliferator-activated receptor *γ* (PPAR*γ*) provided important mechanistic information [26–28]. In this particular case, the fatty acid is bound to the receptor so that the carboxylate and the electron-withdrawing groups (either nitro- or keto-) interact with polar residues in the binding pocket while the aliphatic chain is stabilized through hydrophobic interactions [26–28]. Moreover, it has been proposed that polar side chains close to the electrophilic carbon may enhance the electron-withdrawing effect of the ketogroup thus promoting Michael addition reactions [26].

By covalently reacting with multiple target proteins, electrophilic derivatives of long chain PUFAs activate a complex cascade of signaling events. In addition to the rate of Michael reaction, the biological activity of a given electrophile in the cellular environment will depend on multiple factors, including the reversibility of the covalent binding through beta elimination, intracellular concentration, and levels of glutathione (GSH), glutathione S-transferases, and multidrug resistance proteins [24, 29]. Covalent binding of soft electrophiles to cysteine thiols does not provide a static modification as it reverses via beta elimination and participates to inter- and intramolecular electrophile exchange between thiols. Beta elimination reactions occur via nonenzymatic mechanisms and are favored at high pH values, with the ratio of product to substrate being determined by the equilibrium constant. Enzymatic catalysis of beta elimination reactions has been reported for GS-electrophile conjugates and involves the enzyme glutathione-S-transferase (GST) [30, 31]. Alternatively, the binding of an electrophile with a nucleophilic residue can be reversed through exchange reactions that are favored in the presence of high concentrations of low molecular weight acceptor nucleophiles, such as GSH [32–34]. More recently, it has been proposed that the addition of electrophiles to protein thiols can also be reversed via an enzyme thioredoxin 1 (Trx1) catalysed transalkylation reaction in the presence of GSH [35].

While reversible binding of an electrophilic lipid may represent an important signaling mechanism, irreversible binding and high concentration are generally associated with cytotoxicity due to glutathione depletion, protein misfolding, and irreversible modification of enzyme activities [36]. For example, HNE has been historically viewed as a toxic mediator contributing to oxidative damage related to its elevated concentration found under pathological conditions and the formation of stable, irreversible adducts mainly via Schiff base formation with lysine residues (Figure 1) [37].

## 3. Nonenzymatic Formation of Omega-3 PUFA Electrophilic Derivatives

Nonenzymatic generation of electrophilic derivatives of long chain PUFAs occurs through free-radical-catalyzed lipid peroxidation of both free and esterified fatty acids. Due to their high unsaturation degree, long chain omega-3 PUFAs are highly prone to free-radical-mediated autoxidation generating a wide range of oxidized metabolites including small reactive *α*,*β*-unsaturated aldehydes and electrophilic cyclopentenone isoprostanes (IsoPs) and neuroprostanes (NPs). Lipid autoxidation reactions are triggered when bisallylic hydrogen is abstracted by an initiating free radical species, such as hydroxyl or superoxide radicals whose production is enhanced under conditions of oxidative stress. This reaction generates a lipid radical that rapidly reacts with molecular oxygen to form a peroxyl radical that in turn abstracts hydrogen from an adjacent PUFA. This results in the formation of a lipid hydroperoxide and a new radical species that starts the chain reaction. Since molecular oxygen (O_2_) is required for this propagation phase, lipid peroxidation proceeds at a higher rate in the hydrophobic environment of cell membranes where oxygen concentrates. The hydroperoxide formed during the propagation phase is highly unstable and can be reduced to an alkoxy radical followed by cleavage of the carbon-carbon bond via *β*-scission or can undergo a Hock rearrangement leading to lipid cleavage [38]. Chain breakdown results in the release of short-chain *α*,*β*-unsaturated aldehydes, including 4-hydroxynonenal and 4-hydroxyhexenal (released from omega-6 and omega-3 PUFAs, resp.) [38, 39]. These short-chain, highly reactive compounds are bifunctional molecules that can undergo both Michael addition and Schiff base formation (Figure 1) and are historically viewed as toxic mediators of oxidative stress.

In addition to generating the highly unstable lipid hydroperoxide, lipid peroxyl radicals can undergo endocyclization followed by further addition of molecular oxygen leading to the formation of prostaglandin-like bicyclic endoperoxide intermediates that are further metabolized to IsoPs. Arachidonic acid-derived E_2_ and D_2_-IsoPs readily dehydrate in aqueous solution to cyclopentenone-containing electrophilic A_2_/J_2_-IsoPs [40, 41]. Similarly, autoxidation of EPA and DHA generates electrophilic A_3_/J_3_-IsoPs and A_4_/J_4_-NPs, respectively, which covalently react with target proteins promoting anti-inflammatory and cytoprotective actions [13, 17, 42, 43].

## 4. Enzymatic Generation of **α**,**β**-Unsaturated Ketoderivatives of Omega-3 PUFAs

Several enzymatic mechanisms lead to the formation of oxygenated electrophilic derivatives of omega-3 fatty acids. Three enzyme families are mainly responsible for the oxygenation of omega-3 PUFAs, namely, cyclooxygenases (Cox), lipoxygenases (LOs), and cytochromes P450 [10, 11, 44, 45]. By acting alone or in concerted transcellular biosynthetic mechanisms, these enzymes generate epoxy- as well as mono-, di-, and three-hydroxyl species that can be further oxidized to electrophilic *α*,*β*-unsaturated keto-derivatives by cellular dehydrogenases. The pattern of oxidized lipids released by a given cell type will depend on substrate availability, enzyme expression and activation state, and overall oxidative status. For example, the expression of Cox-2 is controlled at the transcriptional level and is induced during inflammation [46]. In contrast, 5-LO is constitutively expressed and its activity depends on the translocation to the nuclear membrane, association with 5-LO activating protein (FLAP), and access to substrate [47]. In addition to these mechanisms, the activity of these enzymes is modulated by the lipid peroxide tone which in turn depends on the oxidative status of the cell [48]. Similarly, the activity of dehydrogenase enzymes, including 5-hydroxyeicosanoid dehydrogenase (5-HEDH) and 15-hydroxyprostaglandin dehydrogenase (15-PGDH), depends on the availability of the cofactor NAD(P)+ which increases after exposure of cells to oxidative stress or, in phagocytic cells, during the activation of respiratory burst [49–51].

**Figure 2:**
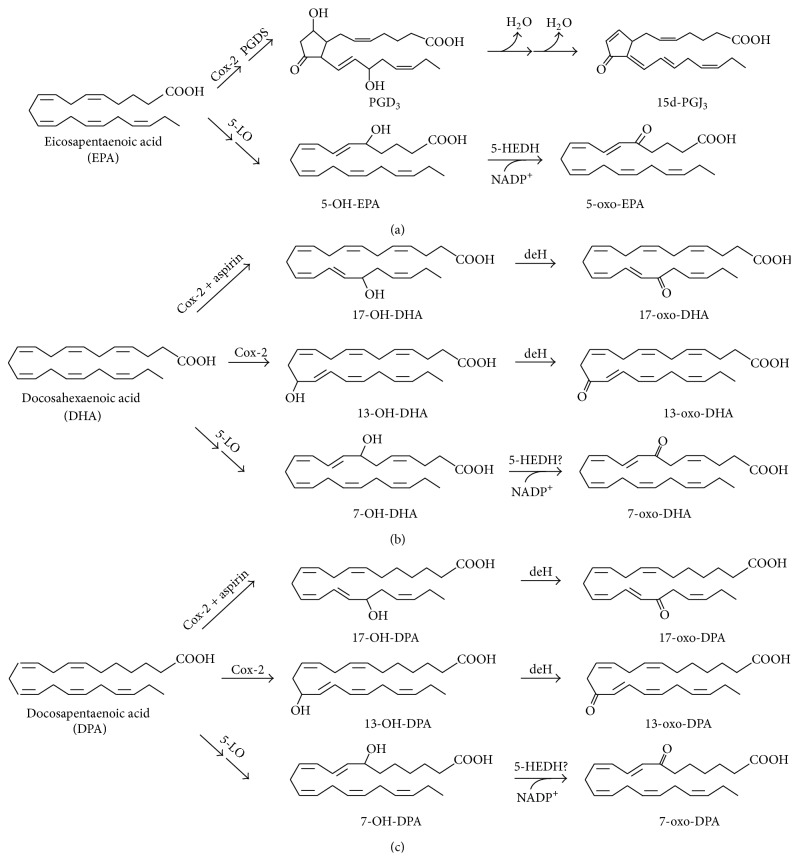
Enzymatic generation of electrophilic ketoderivatives of EPA (a), DHA (b), and DPA (c). Cox-2, cyclooxygenase-2; PGDS, prostaglandin D synthase; 5-LO, 5-lipoxygenase; 5-HEDH, 5-hydroxyeicosanoid dehydrogenase; deH, cellular dehydrogenases.

Endogenous generation of electrophilic *α*,*β*-unsaturated derivatives of omega-3 PUFAs has been reported in several cell types. In activated macrophages, 13-oxo-derivatives of DHA and DPA are formed in two enzymatic steps involving Cox-2 and a cellular dehydrogenase. In the presence of aspirin, Cox-2 converts DHA and DPA into 17-OH-derivatives which are then oxidized to 17-oxo-DHA and 17-oxo-DPA (Figure 2) [14]. Primary alveolar epithelial cells (AEC) supplemented with DHA generate the electrophilic 14-oxo-DHA via a 15-PGDH dependent mechanism [52]. These electrophilic compounds display anti-inflammatory and cytoprotective properties [12, 14]. When using EPA as substrate, Cox-2 catalyses the conversion of this omega-3 PUFA into PGH_3_ which is further metabolized to 3-series prostaglandins. In aqueous environment, PGD_3_ undergoes two nonenzymatic dehydration steps to give the electrophilic cyclopentenone-containing 15d-PGJ_3_ (Figure 2) [15, 16]. In human neutrophils, 5-LO-dependent generation of electrophilic 5-oxo-EPA, 7-oxo-DHA, and 7-oxo-DPA has been reported to be increased upon dietary supplementation with the precursors DHA and EPA (Figure 2) providing evidence that endogenous generation of electrophilic derivatives of omega-3 PUFAs can be effectively modulated through dietary interventions [9, 53].

## 5. Electrophile-Sensitive Signaling Pathways

Electrophilic derivatives of long chain PUFAs promote cytoprotective and anti-inflammatory actions by covalently and reversibly adducting to target proteins inducing a complex cascade of cytoprotective signaling events. Growing evidence supports that the beneficial actions of dietary omega-3 PUFAs are partly mediated by their electrophilic oxygenated derivatives. The Nrf2-dependent antioxidant response, the heat shock response, the NF-*κ*B inflammatory pathway, and the PPAR*γ* are among the most studied pathways regulated by electrophiles and participate in transducing the beneficial actions of electrophilic omega-3 PUFAs. Recently, growing evidence supports that electrophilic lipids also contribute to epigenetic control of gene expression through direct binding to histones or histone-modulating enzymes and through regulating microRNA expression.

### 5.1. Nuclear Factor Erythroid 2-Related Factor 2 (Nrf2) and Its Inhibitor Kelch-Like ECH-Associated Protein 1 (Keap1)

**Figure 3:**
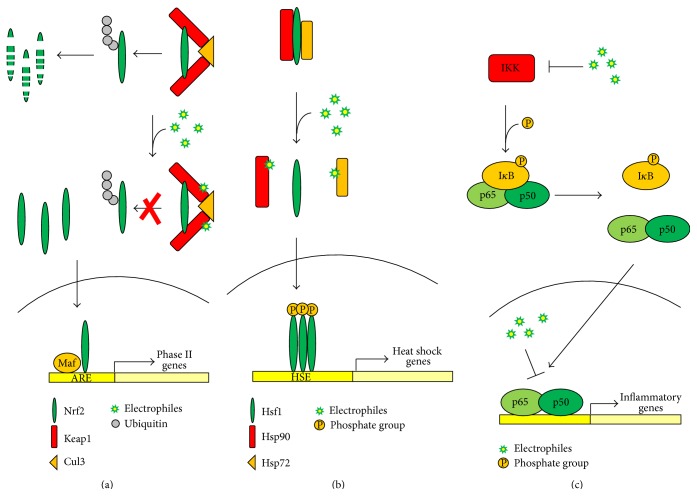
Signaling pathways modulated by electrophilic lipids. (a) Nrf2-dependent antioxidant response. Under basal conditions, Keap1 binds to Nrf2, sending it to proteasomal degradation via cullin-3-dependent ubiquitination. Electrophilic lipids react with target cysteines on Keap1 inhibiting ubiquitination of Nrf2 and promoting nuclear accumulation of Nrf2 which leads to the activation of ARE-dependent genes; (b) Heat shock response. Under basal conditions, inert Hsf1 is found in the cytoplasm bound with Hsp72 and Hsp90. Electrophiles react with Hsp72 and Hsp90 and promote Hsf1 release, phosphorylation, trimerization, and translocation to the nucleus where Hsf1 activates the transcription of heat shock response genes. (c) NF-*κ*B pathway. Under basal conditions, IKK phosphorylates I*κ*B, causing the release of the heterodimer p50/p65 which translocates to the nucleus and activates a variety of proinflammatory mediators. Electrophiles react with IKK leading to kinase inhibition and blocking NF-*κ*B activation. In addition, covalent reaction of electrophiles with target cysteines in the DNA-binding domain of p65 and p50 inhibits their binding to DNA.

Cells are equipped with highly efficient protective mechanisms to overcome chemical and oxidative insults. These include a large number of detoxification proteins such as phase II enzymes, like NAD(P)H:quinone oxidoreductase 1 (NQO1), the enzymes required for the synthesis and metabolism of glutathione, and the heme oxygenase 1 (HO-1). The expression of these proteins is controlled at their transcriptional level and depends on the presence of a cis-acting promoter element called the antioxidant or electrophile responsive element (ARE/EpRE) which is specifically recognized by the transcriptional factor Nrf2, the master regulator of the inducible antioxidant response [54]. Under basal conditions, Nrf2 binds to its negative regulator, Keap1, an adaptor for the ubiquitin ligase Cul3, which targets Nrf2 to ubiquitination and proteasomal degradation. In response to electrophilic inducers, Keap1-mediated ubiquitination of Nrf2 is inhibited and de novo synthesized Nrf2 protein accumulates in the nucleus [55, 56]. Upon nuclear translocation, Nrf2 forms heterodimers with small Maf proteins and recruits other transcriptional factors required for the activation of ARE elements thus starting its transcriptional program (Figure 3(a)). In human Keap1, Cys-273 and Cys-288 located in the intervening region (IVR) are crucial for basal turnover of Nrf2 [56, 57]. The highly reactive cysteine at position Cys-151, which is 100% conserved between species, appears to be critical for a subset of Nrf2 activators, including the electrophilic sulforaphane and 4-HNE [57, 58]. Cysteine 151 is located in the N-terminal BTB domain of Keap1 which is required for proper interaction with Cul3 [57, 59]. Covalent adduction of electrophiles to Cys-151 inhibits Keap1-mediated ubiquitination of Nrf2 leading to stabilization and nuclear accumulation of newly synthesized protein [56]. The electrophilic Nrf2-inducers 15d-PGJ_2_ and nitrofatty acids primarily form adducts with Cys-273 and Cys-288 on Keap1, displaying a much lower reactivity towards Cys-151, suggesting that different patterns of cysteine modification can lead to Keap1 inhibition and Nrf2 activation [60, 61]. Nuclear accumulation of Nrf2 and induction of its target genes have been reported in different experimental models in response to several omega-3 PUFAs derived electrophiles, including the DHA-derivatives 4-HHE and 17-oxo-DHA, the DPA-derivative 17-oxo-DPA, and the EPA-derivatives A_3_/J_3_-IsoPs [12–14, 62].

Multiple alterations of the Nrf2 pathways have been associated with the development and progression of chronic disorders. For example, a mutation of the gene DJ-1, encoding a positive regulator of Nrf2, leads to development of a monogenic form of Parkinson's disease (PD) [63, 64]. Decline of Nrf2 expression has been reported in the lung of chronic obstructive pulmonary disease (COPD) patients [65] and dysfunction of Nrf2 has been correlated with severe asthma in children [66]. In mice exposed to cigarette smoke, disruption of Nrf2 enhanced the susceptibility to emphysema, increased neutrophils influx to the lung, and decreased the expression and activity of HDAC2 thus enhancing oxidative stress-induced inflammation and contributing to steroid resistance [67, 68]. In murine models of asthma, Nrf2 deficiency has been associated with increased eosinophils infiltration into the lungs and enhanced severity of the asthmatic response due to the reduced expression of the antioxidant genes [69].

Based on the growing evidences supporting the central role of Nrf2 in controlling the oxidative status of the cell and the inflammatory response, there has been a growing interest towards the development of new small molecules activators of Nrf2 as drugs for chronic degenerative disorders [54].

### 5.2. Heat Shock Response

Heat, oxidative stress, and other cellular insults induce the heat shock response (HSR) that protect the cell from misfolded and aggregated protein damage through the induction of a large family of genes encoding factors involved in protein synthesis, folding, trafficking, and degradation [70]. This response is mainly controlled at the transcriptional level and depends on the activity of a family of heat shock factors among which Hsf1 is essential for the regulation of heat shock proteins (HSPs) expression. Under homeostatic conditions, Hsf1 is an inactive monomer located in the cytoplasm and bound to the chaperones Hsp70 and Hsp90. Under conditions of heat shock and oxidative stress or in presence of electrophilic inducers, Hsp90 and Hsp70 dissociate from Hsf1. Once released, Hsf1 undergoes multistep processing involving phosphorylation, nuclear translocation, trimerization, and binding to the heat shock elements (HSE) ultimately leading to transcriptional activation of the heat shock genes [70] (Figure 3(b)).

It has been shown that 4-HNE and the mild electrophile sulphoxythiocarbamate alkyne (STCA) form stable adducts with Hsp90 and Hsp72 (the inducible form of Hsp70) [71, 72]. More specifically, STCA covalently reacts with Cys-412, Cys-564, and Cys-589 (or Cys-590) in human recombinant Hsp90, most likely impairing its chaperone activity, leading to Hsf1 release and HSR activation [72]. In a rat model of ethanol-induced oxidative stress, covalent reaction of 4-HNE with the Cys-267 in the ATPase domain of Hsp72 and with Hsp90 Cys-572 has been reported leading to a reduced chaperone activity [73, 74]. Although the complete pattern of adduction by electrophilic lipids has not been clearly identified and the molecular mechanisms of action still remain to be defined, induction of the Hsf1-dependent HSR has been reported for several electrophilic derivatives of long chain PUFAs, including nitro-fatty acids (nitro-FAs) and 15d-PGJ_2_ [75–77].

Growing evidence indicates that electrophilic inducers of the Nrf2 pathway are also activators of the heat shock response through covalent binding with Hsp90 and Hsp72 and activation of Hsf1 [72, 78–80]. This supports a model in which the two most prominent cellular cytoprotective pathways, namely, the Nrf2-dependent antioxidant response and the HSR, can be induced through similar pharmacological mechanisms within a common regulatory network.

Since the induction of the HSR plays a central role in protecting the cell from external insults and protein damage, the sensitivity of this pathway to activation by electrophilic species further supports that some of the beneficial actions that have been associated with this class of compounds rely on the activation of this specific pathway.

### 5.3. Nuclear Factor-Kappa B (NF-*κ*B) Proinflammatory Pathway

Inhibition of the NF-*κ*B proinflammatory pathway is one of the best-defined mechanisms through which PUFAs electrophilic derivatives promote anti-inflammatory and cytoprotective actions. The NF-*κ*B signaling pathway controls the onset of innate and adaptive immune response by activating the expression of cytokines, adhesion molecules, proinflammatory enzymes, and transmembrane receptors in response to several stimuli. The activity of this transcriptional factor is controlled at multiple levels and electrophilic PUFAs have been reported to interfere with most of them.

In unstimulated conditions, NF-*κ*B resides in the cytoplasm, mostly as a heterodimer composed of p65 and p50 and bound to the inhibitor I*κ*B. In response to proinflammatory stimuli, the complex I*κ*B kinase (IKK) becomes activated by phosphorylation and in turn phosphorylates I*κ*B, sending it to ubiquitination and proteasomal degradation. This results in the release and nuclear translocation of NF-*κ*B, leading to transcriptional activation of its target genes [81] (Figure 3(c)).

A well-described inhibition mechanism of NF-*κ*B by electrophiles occurs through covalent binding to the highly conserved cysteines at position Cys-38 of p65 and Cys-62 of p50, within their DNA-binding domain. More specifically, 15d-PGJ_2_ and nitro-FAs covalently react with these residues leading to the loss of DNA binding activity [82–85]. The electrophilic DHA and DPA derivatives, 17-oxo-DHA and 17-oxo-DPA, also suppress p65 DNA binding activity, although the precise mechanism of action has not been established yet [14]. Inhibition of the IKK kinase by alkylation of Cys-179, located in the activation loop of IKK*β*, represents an alternative mechanism through which electrophiles suppress the NF-*κ*B pathway. This modification, which has been demonstrated for the DHA-derived cyclopentenone-NPs and for 15d-PGJ_2_, results in the suppression of kinase activity, I*κ*B stabilization, and consequent NF-*κ*B inhibition [17, 84, 86]. More recently, an additional mechanism has been reported through which electrophilic nitro-FAs can suppress the activation of this proinflammatory pathway, that is, by interfering with the recruitment into lipid rafts of the signaling mediators required for triggering the NF-*κ*B pathway [87]. Since alterations of lipid-raft-related proinflammatory signaling have been identified as a mechanism through which omega-3 PUFAs exert anti-inflammatory actions, bioactive electrophilic derivatives provide an alternative mechanism to the reported changes in membrane composition and fluidity [6, 7].

Persistent activation of the NF-*κ*B pathway represents a common feature of virtually all chronic diseases, including neurodegenerative disorders, asthma, and COPD. Increased nuclear accumulation of NF-*κ*B has been measured in the Parkinsonian brain as well as in neurons and peripheral blood mononuclear cells (PBMCs) of Alzheimer's patients, and the neurotoxic amyloid-beta (A*β*) peptide is a strong inducer of the NF-*κ*B [88–90]. Dysregulation of this pathway has been reported in asthmatic patients and in lungs of COPD subjects, where cigarette smoke contributes to persistent activation of the NF-*κ*B [91–93]. In these cases, and for most of the chronic inflammatory disorders, targeting the NF-*κ*B through pharmacological approaches appears to be a promising therapeutic strategy. In this regard, electrophilic lipids represent a class of compounds with a great pharmacological potential.

### 5.4. Peroxisome Proliferator-Activated Receptor *γ* (PPAR*γ*)

The PPAR*γ* is a member of the nuclear hormone receptor superfamily of transcription factors that is highly expressed in adipose tissue, macrophages, and dendritic cells (DCs) [94]. Upon ligand binding, the PPAR*γ* forms a heterodimer with the retinoid X receptors (RXRs), binds to PPAR*γ* response element (PPRE), and recruits transcriptional coregulators that control the expression of genes involved in adipogenesis, glucose metabolism, and macrophage and DCs function [95]. Its association with coregulatory proteins occurs through interactions with the surface of the ligand binding domain (LBD) and is controlled by the conformational changes induced by ligands ultimately modulating gene expression [95]. The PPAR*γ* LBD is a hydrophobic pocket that can accommodate a wide range of lipophilic ligands, including long chain PUFAs and oxidized fatty acids. The presence of a reactive cysteine within the LBD (Cys-285) confers a special sensitivity for electrophilic lipids, which are best described as partial agonists and are able to covalently bind to PPAR*γ* [26]. This provides evidence for the particular activation by electrophiles, resulting in activation at lower concentrations, and for prolonged periods of time when compared to nonelectrophilic PUFAs [26]. Covalent addition to Cys-285 and activation of the PPAR*γ* have been reported for nitro-FAs, oxo-ETEs, 15d-PGJ_2_ and for the electrophilic omega-3 PUFA derivatives 4-oxo-DHA, 17-oxo-DHA, 17-oxo-DPA, and 15d-PGJ_3_ [14, 16, 26, 28, 96, 97]. The activation of the PPAR*γ* produces a cascade of events that differ based on the cell type and condition ranging from antidiabetic to neuroprotective, anti-inflammatory, and cardioprotective actions [96, 98–103]. For example, in murine model of diabetes, activation of PPAR*γ* by nitro-FAs restored insulin sensitivity and blood glucose levels [96] and in experimental models of COPD, treatment with PPAR*γ* agonists contrasted cigarette smoke-induced inflammation and downregulation of HDAC2 [93, 102, 103]. Overall, there is increasing evidence that activating the PPAR*γ* promotes beneficial effects in several pathological conditions. As potent activators of this transcriptional factor, electrophilic PUFAs enhance PPAR*γ*-dependent signaling which becomes part of the complex salutary cascade of events triggered by these lipid derivatives. However, because of the complex network of signaling pathways that are activated in response to electrophilic PUFAs, it is still a challenge to define to what extent the PPAR*γ* is responsible for the observed effects.

### 5.5. Epigenetic Modulation by Electrophiles

Epigenetic control of gene expression involves DNA, RNA, and protein modification as it occurs during DNA methylation, covalent modification of histones, and posttranscriptional regulation of gene expression by noncoding microRNAs (miRNAs) [104, 105]. Growing evidence supports that electrophilic lipids participate in epigenetic mechanisms at multiple levels, that is, by directly adducting histones, by regulating the activity of histone-modifying and DNA methylating enzymes, and by controlling miRNA expression.

Histones are lysine- and histidine-rich proteins that are required for the control of chromatin structure. A recent study has shown that, under physiological conditions, the electrophilic 4-ONE covalently adds to histones H2A (His-123), H2B (His-82, His-109, Lys-116), H3 (Lys-23, Lys-27), and H4 (Lys-79) [106]. Interestingly, modifications of H3 Lys-23 and Lys-27 (known sites of acetylation and methylation) interfered with the process of nucleosome assembly. These findings support that electrophilic adduction to histones is a mechanism through which these reactive species control gene expression under physiological and pathological conditions.

While limited reports are available on histone adduction by electrophiles, more data exist on electrophilic modification of histone-modifying enzymes, including histone deacetylases (HDACs) and acetyl transferases (HATs) [107–111]. In this regard, electrophilic lipids containing an *α*,*β*-unsaturated carbonyl moiety covalently bind to two highly conserved cysteines that are present in class I histone deacetylases HDAC1, HDAC2 and HDAC3, namely, Cys-261 and Cys-273 in HDAC1 [108, 109]. These modifications disrupt the interaction of histones with their substrate and reduce their enzymatic activity. Similarly, 15d-PGJ_2_ was shown to inhibit the activity of the histone deacetylase Sirt1 due to its electrophilic carbon [107]. The p300 HAT is also a target for electrophilic addition by 15d-PGJ_2_. More specifically, it has been shown that 15d-PGJ_2_ undergoes Michael addition with the catalytic cysteine at position 1438, within the substrate binding site of p300, leading to inhibition of its enzymatic activity [111]. The enzyme DNA methyltransferase 1 (DNMT1) possesses a reactive catalytic cysteine at position 1226 that covalently reacts with soft electrophiles with Michael addition. This reaction was characterized for curcumin, a dietary electrophile with protective anti-inflammatory actions, and leads to inhibition of enzyme activity resulting in DNA hypomethylation [112]. Finally, it has been reported that electrophiles can modulate the expression of several miRNAs thus providing an additional mechanism through which these reactive species participate in controlling gene expression via epigenetic mechanisms [113, 114].

Several data support that omega-3 PUFAs contribute to epigenetic control of gene expression. Dietary supplementation of omega-3 PUFAs has been correlated with reduced histone acetylation levels, changes in histone methylation/phosphorylation status, and modification of global DNA methylation [115–118]. Also, it has been reported that the oxygenated derivative of DHA, Resolvin D1, is able to modulate the expression of specific miRNAs [119]. It remains to be established whether these effects are mediated by electrophilic derivatives of omega-3 PUFAs.

## 6. Therapeutic Potential of Electrophilic Derivatives of Omega-3 PUFAs

In recent years, a growing number of electrophilic drugs have entered clinical development. The interest towards this class of compounds for drug development relies on their ability to simultaneously activate multiple antioxidant and cytoprotective pathways that are involved in the pathophysiology of several diseases where inflammation and oxidative stress play a central role. The therapeutic potential of the naturally occurring electrophile sulforaphane has been investigated in several clinical trials for diseases ranging from cancer to diabetes and COPD [120–123]. Very recently, the Food and Drug Administration (FDA) has approved the use of the electrophilic dimethyl fumarate for the treatment of relapsing forms of multiple sclerosis [124]. Until now, the activity of electrophilic derivatives of long chain PUFAs such as nitro-FAs and cyclopentenone prostaglandins has been assessed in preclinical models, including* in vitro* and* ex vivo* studies and animal models of disease. In murine models, nitro-FAs displayed antidiabetic actions, reduced vascular inflammation, attenuated hypoxia-induced pulmonary hypertension, and reduced the severity of allergic airways disease being more effective than fluticasone propionate in contrasting neutrophilic inflammation [87, 96, 125, 126].

Increasing evidence shows that oxygenated derivatives of omega-3 PUFAs transduce the beneficial effects that have been associated with DHA and EPA dietary intake. Several reports have demonstrated the protective effects of mono-, di-, and trihydroxyl derivatives of DHA and EPA in murine models of disease [127, 128]. However, preclinical data on the therapeutic actions of omega-3 PUFAs electrophilic oxo-derivatives remain very limited. In murine models of leukemia, the EPA metabolite 15d-PGJ_3_ selectively targeted leukemia stem cells (LSCs) for apoptosis in the spleen and bone marrow, displaying superior performance compared to available chemotherapeutic approaches [15]. Furthermore, the anti-inflammatory and cytoprotective actions of 13- and 17-oxo-DHA, A_4_/J_4_-NPs and 15d-PGJ_3_ have been demonstrated in several* in vitro* models of disorders ranging from neurodegeneration to airways inflammatory diseases [12, 14, 16, 17].

The use of endogenous omega-3 PUFAs electrophilic derivatives as drugs would offer several advantages including the possibility to increase their concentration via two routes, that is, by oral supplementation of the fatty acid precursor and by direct administration of the electrophilic compound [8–11]. In the specific case of airways inflammatory disorders, a further advantage is correlated to the possibility of using these compounds for inhalation therapy as recently reported for Resolvin D1 [127]. In this regard, recently published data suggest that, in presence of oxidative stress, as it occurs in the lung of COPD patients, hydroxyl derivatives of DHA, including Resolvin D1, may be further oxidized to electrophilic ketoderivatives which could be the final mediators of the observed beneficial actions [12]. Regarding the possibility of enhancing the formation of electrophilic derivatives of omega-3 PUFAs through dietary administration of their fatty acid precursors, therapeutic doses should be carefully evaluated. Recent reports suggest that high intake of omega-3 PUFAs may be not without risk. In fact, high doses of omega-3 PUFAs may dampen the immune system altering pathogen clearance or interfere with tumor surveillance mechanisms thus leading to adverse outcomes [129, 130].

Finally, when considering direct administration of electrophilic drugs, important factors should be taken into consideration, which are related to the typical reactivity of these compounds. In fact, electrophiles covalently react with target cysteines and their signaling can accumulate over time. To determine bioavailability and pharmacokinetics of these compounds, classical pharmacological methods are not applicable calling for the development of new approaches. Furthermore, high doses of electrophilic inducers of Nrf2 may promote cancer cell proliferation and chemoresistance in the long run [131]. To better evaluate this and other potential toxic effects, well-designed long-term clinical trials should be conducted.

Overall, growing evidence supports that electrophilic oxo-derivatives of omega-3 PUFAs promote the beneficial effects that are observed upon dietary supplementation of these fatty acids. However, research aiming at translating these findings into new therapeutic applications is still at the beginning and preclinical and clinical studies should be conducted to assess the potential of these compounds as drugs for the treatment of inflammatory disorders.
